# Collaborative research networks in health: a pragmatic scoping study for the development of an imaging network

**DOI:** 10.1186/s12961-015-0067-y

**Published:** 2015-12-09

**Authors:** Tracy Elizabeth Robinson, Nicole Rankin, Anna Janssen, Deborah Mcgregor, Stuart Grieve, Timothy Shaw

**Affiliations:** Discipline of Nursing and Midwifery, University of Canberra, Canberra, Australia; Sydney West Translational Cancer Research Centre, Westmead Hospital, Sydney, Australia; Sydney Catalyst Translational Research Fellow, The University of Sydney, Sydney, Australia; Research in Implementation Science and eHealth, Faculty of Health Sciences, University of Sydney, Sydney, Australia; Sydney Translational Imaging Laboratory, Heart Research Institute & Charles Perkins Centre, University of Sydney, Sydney, Australia; Department of Radiology, Royal Prince Alfred Hospital, Sydney, Australia

**Keywords:** Collaborative research networks, Enabling factors, Translational research

## Abstract

**Background:**

Collaborative research networks are often touted as a solution for enhancing the translation of knowledge, but questions remain about how to evaluate their impact on health service delivery. This pragmatic scoping study explored the enabling factors for developing and supporting a collaborative imaging network in a metropolitan university in Australia.

**Methods:**

An advisory group was established to provide governance and to identify key informants and participants. Focus group discussions (n = 2) and semi-structured interviews (n = 22) were facilitated with representatives from a broad range of disciplines. In addition, a survey, a review of relevant websites (n = 15) and a broad review of the literature were undertaken to elicit information on collaborative research networks and perceived needs and factors that would support their involvement in a multi-disciplinary collaborative research network. Findings were de-identified and broad themes were identified.

**Results:**

Participants identified human factors as having priority for developing and sustaining a collaborative research network. In particular, leadership, a shared vision and a communication plan that includes social media were identified as crucial for sustaining an imaging network in health research. It is important to develop metrics that map relationships between network members and the role that communication tools can contribute to this process.

**Conclusions:**

This study confirms that human factors remain significant across a range of collaborative endeavours. The use of focus group discussions, interviews, and literature and website reviews means we can now strongly recommend the primacy of human factors. More work is needed to identify how the network operates and what specific indicators or metrics help build the capacity of clinicians and scientists to participate in translational research.

## Background

The Australian health sector landscape is undergoing a dramatic transformation in response to rapid epidemiological and demographic changes, including an aging population, increased life expectancy, and higher levels of chronic illness [1]. In particular, chronic health conditions and new medical technologies have contributed to an increasing burden on an already costly healthcare system [2]. At the forefront of Australian strategic health priorities are chronic diseases such as cancer, diabetes, cardiovascular disease and mental health, all of which have a high disease burden [2]. A high prevalence of multi-morbidities in aging populations also results in a greater demand to integrate and coordinate these priority areas in order to improve patient outcomes [3]. This means the health workforce, researchers and policymakers in Australia are increasingly required to undertake collaboration and networking outside their own disciplines to identify creative solutions to complex problems.

In addition to collaboration and networking, there is a need for greater efficiency in health research, with an imperative to reduce the time between new scientific discoveries and patient benefits [4–6]. It has been estimated that new discoveries take an average of 17 years to be translated into clinical practice [7]. Hence, the translation of research evidence into practice is critical and governments and funding bodies in Australia are increasingly focussing on ways to encourage knowledge transfer from ‘bench to bedside’. Collaborative research networks have been touted as a solution for enhancing knowledge translation [8], but questions remain about how to evaluate their impact on health service delivery [9]. Definitions of collaborative research networks differ, but they commonly identify relationships, resources and knowledge transfer as key features of networks that *“…involve the creation, combination, exchange, transformation, absorption, and exploitation of resources…within a wide range of formal and informal relationships*” ([8], p. 21). This highlights the importance of relationships in sustaining networks, but we cannot assume that simply establishing a collaborative network as a structural solution will inevitably result in improved collaboration [9].

Recently, the Australian Government published ‘The Strategic Review of Health and Medical Research in Australia’ (the McKeon Review) [10], with the goal of building the capacity of medical research to improve the effectiveness and efficiency of the health system. This report recommends that healthcare jurisdictions should utilise research clusters as a catalyst for multidisciplinary team building in order to facilitate more rapid exchange of information and new evidence [10]. Collaborative research networks have the potential to translate knowledge across the research, policy and practice divides – domains where collaboration is often constrained by different priorities and languages [11,12].

In response to the McKeon Review and the changing expectations of Government and research funders, the University of Sydney initiated its own health and medical research strategic review – the Wills Review [13]. A primary focus of the Wills Review was to identify strategies for the University that would facilitate the development and support of collaborative research networks that are consistent with the new expectations of governments and funding bodies [13]. The University of Sydney has subsequently implemented a number of strategies to promote collaborative research activity, including the establishment of the Charles Perkins Centre (CPC) and the Sydney Research Networks Scheme. These initiatives are important because the Wills Review also identified that health and medical disciplines are often fragmented by both geography and organisational structures [13]. The CPC was established by the University to encourage the growth of collaborative, interdisciplinary research and education structures to address the burden of chronic disease, including obesity, diabetes and cardiovascular disease. In order to foster an integrated approach, the CPC has a focus on translational health research and houses a combination of wet and dry laboratory spaces, new imaging and flow cytometry facilities, including live cell imaging, clinical research facilities and a biobank of specimens. Hence, the colocation of these facilities makes the CPC an ideal context for capacity building in multidisciplinary research.

Key enabling factors for the development of collaborative networks in health have been identified, including knowledge sharing, a positive social climate and strong co-worker ties [14]. In particular, knowledge sharing is important and strategies that support the translation of knowledge in networks have been categorised as having push, pull and exchange components [15]. Push efforts include the distribution of knowledge, while pull efforts include employing knowledge brokers and social media spaces that encourage participation in the network [15]. Exchange efforts include the establishment of formal partnerships and policies to support evidence-informed decision making [15]. The development of online resources such as dedicated ‘knowledge portals’ and online collaborative networks can facilitate push, pull and exchange activities and key stakeholders need to be actively involved in their development. Despite agreement that collaborative networks seem to be the best method to address the complexity and fragmentation inherent in health research, the manifold conceptual models and definitions of translational research mean that collaborative networks are difficult to establish and evaluate [16].

In 2012, the University of Sydney commissioned its Workforce Education and Development Group to scope the development of an imaging network at the CPC. The scoping study was used to garner information for the design of a web portal to support the network and to identify potential partners and strategies for translating knowledge across the network. A number of medical imaging networks have been formed in Australia (in both the university and private sectors), but the authors found no published studies that explored their scope or enabling factors for translational research.

Given the paucity of literature on how to sustain health research networks and how to progress knowledge translation across disciplines in health networks, it is important to engage with clinicians to bridge gaps between discovery and implementation [17] and to identify methods and measures for evaluating ‘team science’ [18]. In addition, the literature identifies an inherent tension between the opportunities for innovation that collaborative research networks generate and the additional costs involved in creating and sustaining collaborative enterprises [19]. Hence, there was a strong rationale for scoping the contextual and implementation issues involved in establishing and sustaining a collaborative research network in medical imaging. This paper reports on the findings from a pragmatic scoping study that aimed to identify effective web-based supports for researchers in a collaborative imaging network and optimum strategies for promoting translational research across the network at the University of Sydney, Australia. This included the identification of participant’s current research collaborations, perceived needs, barriers and enabling factors for their involvement in a multidisciplinary collaborative imaging network.

## Methods

**Figure 1:**
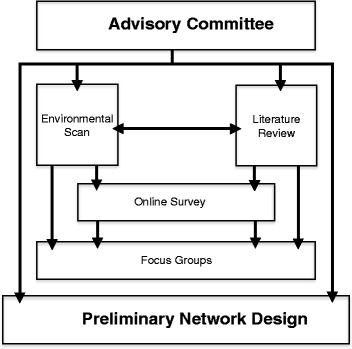
**Study methods.**

An expert advisory group was convened in order to engage with and ensure the involvement of all key stakeholders in the consultation process. This group comprised representatives from the CPC, Faculty members (who also worked as heads of imaging), representatives from the Research Office and from the Brain and Mind Research Institute at the University of Sydney. The advisory group provided governance for the study and assisted with the identification of key informants for semi-structured interviews and focus group discussions. Consultation with key stakeholders adds methodological rigour to scoping studies and should be seen as an essential component of this approach, despite challenges in incorporating the findings from consultations in study results [20]. In addition, scoping studies aim to summarise breadth of evidence and therefore require a broader focus that is often well suited to qualitative analytical techniques [20]. Because the current study focused on exploring a range of participant experiences and the factors that impact on their collaborative research endeavours, a multi-methods approach was utilised. Figure 1 provides an overview of how the multi-methods were combined and utilised in this study.

The multi-method approach included (1) an environmental scan of existing online collaborative networks; (2) a brief online survey that was emailed to participants to explore available imaging resources and rank components of a proposed web site (the brief survey was also administered to focus group and interview participants); (3) a review of the literature describing enabling factors for the development and sustainability of collaborative research networks that informed the development of focus group and interview questions; and (4) focus group discussions and semi-structured interviews with a range of potential network stakeholders. These methods are explained in more detail below.

### Environmental scan of web-based collaborative networks

A review was undertaken of relevant websites with the primary purpose of identifying factors for engaging and connecting a collaborative network. Inclusion criteria were existing University of Sydney networks and international university and non-university health networks. While imaging sites were of specific interest, the search strategy did not exclude non-imaging sites. After a broad review, a comprehensive assessment was conducted on a refined list of network sites for the purpose of identifying key website features that were included in the brief survey.

### Brief surveys

A 10-question imaging capability assessment survey was developed and disseminated to faculty members by email. The survey used a combination of multiple choice, Likert scale and free-text responses to collect data from participants. Participants were asked to indicate their current research area, how they currently accessed imaging technology and how they rated a range of resources. The survey was emailed to potential users of the network identified by the advisory group. An adapted version of the survey was also offered to focus group and interview participants. This included 17 potential website features that participants were asked to rank on a Likert scale from 1 (not important) to 5 (very important).

### Review of published literature

A broad review of the published literature was undertaken to inform the focus group and interview questions in relation to the main challenges in establishing and maintaining collaborative networks. Data bases from Health (Medline, Embase), Business and Information Technology (Proquest, InFORMIT, Web of Knowledge and Business Source Premier) were reviewed. Because the search terms were broad (“collaborative research networks” and “imaging networks”) only studies from the year 2000 onwards and only those that addressed the establishment and maintenance of collaborative research networks and centres were included in the review.

### Interviews and focus group discussions

Focus group discussions and targeted stakeholder semi-structured interviews were conducted to further explore factors that would enable participation in collaborative research and the imaging networks. Participants were asked to identify their current research collaborations and any modifiable factors that impact on their research activities. Participants were also asked to identify their perceived needs and enabling factors and barriers to their involvement in a multidisciplinary collaborative research network. At the conclusion of each focus group, participants were asked to complete the 17-item survey and rank features for the online imaging portal. In addition, semi-structured interviews were conducted with imaging scientists and clinicians from 13 relevant target groups and organisations. Internal networks and advisory group members nominated key interview informants and flyers were also distributed at three sites to encourage early career researcher participation in focus group discussions and interviews.

The findings from focus group discussions and semi-structured interviews were audio-taped, de-identified, transcribed, and then compared and contrasted. Broad themes were identified and cross checked by a second researcher. A summary of findings was distributed to participants and informed the development of a web portal to enhance collaboration across the network. Permission to conduct this study was received from the University of Sydney Human Research Ethics Committee (No: 2012/394).

## Results

### Environmental scan of web-based collaborative networks

A comprehensive assessment of 15 relevant websites included both university and non-university sites. Most of the websites examined could be described as brochure sites whose primary function is the provision of information, including member profiles, funding and grant opportunities, news and events, and resources. There were no online activities or collaboration opportunities available on any of the university sites reviewed. Similarly, few non-university sites offered online collaboration activities, although the NHS Education for Scotland was a notable exception. This site provides members with their own personal web space to create their content and also offers community tools, including wikis, blogs, discussion forums, tagging and personal profiles to enhance shared learning.

### Survey findings

A total of 51 survey responses were received by mail from a broad range of faculty members, including Engineering and Information Technologies (n = 7), Health Sciences (n = 6), Science (n = 10), and Medicine (n = 17). Generally, respondents were supportive of an online biomedical imaging portal and perceived it as a valuable resource, with many saying they would use such a resource (n = 20). Outlying responses included the crucial role of administrative and technical support (n = 2) and potential benefits in using the portal for booking equipment or obtaining information on where it was located (n = 3). There was some interest in using the online portal for image processing (n = 2) and for developing a forum that facilitates discussion on the application of imaging in research (n = 4). Two respondents identified that they did not support the concept of an online network (n = 2). One participant also commented that there was a low awareness about strategies for translating research into clinical practice and policy and that this was an immediate challenge for the research network.

At the conclusion of the two focus group discussions, 12 participants completed the survey and 11 respondents identified a resources library as important or most important. This was followed by a search engine (n = 9) and an event calendar (n = 9). Low ratings were received for competitions/quizzes (n = 2) and for a bulletin board/marketplace/classifieds (n = 2).

### Themes emerging from the literature and qualitative findings

Two broad themes emerged from the review of the literature and were used to inform the development of focus group (n = 2) and semi-structured interview questions (n = 22). Both technical and human factors have long been cited as crucial for developing and enabling collaborative research networks [21] and were further explored in interviews and focus group discussions. A total of 15 stakeholders participated in two focus group discussions and included scientists, department and laboratory heads, research fellows, clinicians and academics. In addition, 22 semi-structured interviews were conducted with participants from a broad range of disciplines (physics, science, medicine, nursing and health sciences), including imaging scientists, clinicians and early-mid and late career researchers from three sites at the University of Sydney.

The most common theme to emerge from focus group discussions and interviews confirmed the primacy of human factors in establishing and sustaining a collaborative research network. This was seen as particularly important for early career researchers, one of whom noted, “*It’s so important to provide opportunities for junior researchers to interact with experienced researchers and with each other*”. One senior program manager noted that “…*the community needs to exist in the real world before it can take off online*”. Other human factors to emerge include the use of social media and the web portal for finding contacts and potential research collaborators. In addition, participants confirmed the utility of the portal for accessing information about the imaging equipment housed within the university. Only two participants identified some resistance to joining any new online community and one stated that “… *it would have to be offering something special because of the amount of time and effort required*”.

Other human factors identified by participants related to the importance of having clear aims, a vision and influential leadership. As one participant noted, “*We don’t need leaders, but influencers*”. Others stated that there was a need for clinical as well as scientific leaders because some tension between clinical and research leaders was identified by several participants. For example, clinical leaders may receive ‘adjunct’ or conjoint appointments at the university in return for their participation in research, but one respondent stated that these titles “…*no longer have the same cache they once did*”. Hence, the CPC will need to be seen as having “…*an outreach focus rather than an inreach focus*”. Furthermore, because there has not previously been a collaborative research network in imaging at the University of Sydney, several participants perceived this as a challenging enterprise, “…*the question is can you maintain infrastructure with grants*?” In addition, participants commented on the significance of seed funding, even if modest in nature, as a means of promoting participation in collaborative research. They also stated that there was a need for a broad engagement strategy and more interaction between all participating sectors. The importance of human factors was highlighted in the comment that, “*Sharing a document is not the same as having a conversation*”. Hence, the research should drive the technology (not the other way around).

Technical issues identified by participants included security issues and the propriety nature of some data as potential barriers to online collaboration with other disciplines. Participants stated that they would like the online portal to describe and promote imaging equipment and resources as well as provide a collaborative space and online activities to build and support the network. In addition, the need for an online collaborative tool was emphasised. An efficient tool for document sharing is important because, despite the availability of wikis and other university tools, participants reported that sharing documents remains one of their most significant challenges and that current options are not user friendly. This provides further support for the importance of enabling social and collectivist platforms that facilitate sharing of research endeavours and findings.

## Discussion

This study identified a number of key human and technical factors that are vital to support the formation of research networks. Human factors include the value of face to face interaction, having clear aims, a vision and influential leadership, the important role of social media and technology in supporting networks, the value of having seed funding as a means of promoting collaboration, and having a broad engagement strategy across participants and organisations. Technical factors include security issues, the propriety nature of some data, the importance of a document sharing function, and using an online portal to describe and promote imaging equipment and resources.

These findings are consistent with other evaluations of collaborative networks. Williams et al. [6] identified a number of similar human factors impacting on the development of collaborative networks in the context of establishing trust amongst participants. The emphasis placed on face to face contact to build relationships identified in this study supports this focus on building trust amongst participants as a necessary precursor to the development of collaboration. In this study, effective leadership, a communication plan and a shared vision were identified as key enabling factors for collaborative research endeavours. It is important, therefore, that due consideration is given to strategies for strengthening connections and relationships between network participants. For example, network meetings could be organised to bring together champions and researchers, along with service users and funding providers to look critically at the scope of imaging research. This would require administrative support and project oversight that is often overlooked in funding allocations. Research that extends to service delivery and implementation is more challenging and requires particular support, but Thyer [21] notes that interdisciplinary evidence-based practice guidelines are one effective way of enabling stronger links in implementation research.

In the current study, survey results identified communication tools such as wikis, blogs and web-pages as important. Qualitative findings also highlighted the importance of technology-enhanced collaborative tools but evidence in the literature is equivocal and indicates that such tools are not always widely adopted [22]. One enabling factor indicates that people are more inclined to share information when they are engaged and have a role in producing and managing the shared information [23]. The CPC will, therefore, need to support the active participation of members and not rely totally on enhancing access through technology alone. Because information sharing is a collective and social process, further studies would benefit from capturing the nature, operation and relationships between network members and the role that technology and communication tools can play in this process. Carswell et al. [9] recommend using social network analysis to examine partner selection and qualitative approaches to explore network management. This is a useful framework for ongoing evaluation of research networks and highlights the need to identify indicators for how knowledge is translated within networks that was beyond the scope of the present study.

Limitations of the current study include the relatively small number of participants in focus group discussions and the selective nature of the website reviews that did not include a wide range of sites outside the health sector. Although an attempt was made to be broad in terms of the potential network users who were included in the study, it was not possible to collect the views of all potential users and, in particular, no policymakers participated in interviews. Their inclusion would have allowed more understanding of how organisations may embed collaborative research in workplace culture. As the network continues to develop it is vital that consultation with existing and potential network members continues in order to ensure engagement and the development of tools and processes that are seen to be of value to the membership. Furthermore, because scoping studies require a balance between feasibility, significance, and the breadth and comprehensiveness of information that is scoped [20], they are challenging to conduct. Hence, there is a need to clarify what constitutes methodological rigour in scoping studies because they are an increasingly popular option for synthesising health evidence [20]. In the current study, a pragmatic approach was adopted, whereby broad reviews were undertaken and the engagement of an advisory group helped to facilitate ‘buy in’ from a diverse range of health disciplines, but not all potential users were able to be accessed.

Overall, findings from this study highlight the important role of human factors in promoting knowledge translation in collaborative health research that can be readily translated into clinical practice. The CPC offers a rich environment for studying a variety of questions about chronic disease from a range of disciplinary approaches. In addition, because many network members are now co-located, there is an opportunity for ongoing evaluation of exchange activities and discipline linkages that may help to foster translational research to improve outcomes for people with complex and chronic disease. Furthermore, the CPC is well placed to support implementation studies that aim to identify key enabling factors for knowledge translation. This is important because the increasingly competitive nature of health research funding in Australia means that evaluation and implementation scientists face significant hurdles to fund their research, which relies on complex methods and is not always amenable to randomised controlled trials.

Although the current study focused on improving a local network and the findings are not necessarily generalizable, it does provide a more nuanced understanding of the challenges and human factors that enable the development of a collaborative research network in imaging and highlights the need for metrics to capture the relationships and collaborations between network members. Nevertheless, there are ongoing questions about how to sustain the network management and test frameworks for knowledge translation across the relevant policy, practice and research domains. By combining focus group discussions and interviews with a survey and literature and website reviews, we can now strongly recommend the need to address human issues when building a collaborative network. The findings from this study will inform the development of evaluation metrics, including processes for engagement and mapping relationships that will be applied in future multi-disciplinary implementation studies.

## Conclusions

There is wide acknowledgement that creating multi-disciplinary and inter-professional links across networks is important but challenging. Few collaborative research networks have rigorously scoped the context and processes that need to be addressed to initiate and sustain their collaborative research endeavours. The current scoping study used multi-methods to synthesise broad knowledge about how to develop and support a multi-disciplinary imaging research network in the CPC. In particular, it included consultation with key stakeholders, focus group discussions and interviews, which are often omitted from scoping studies. The study has the potential to inform ongoing evaluation of the network, which is essential for identifying how knowledge is translated and the impacts on clinical practice and policy. Relationships and human factors are important enabling factors and future research efforts should be directed to the validation of frameworks that incorporate metrics for key stakeholder engagement and metrics for capturing changing patterns of relationships between network users.

## References

[1] Duckett SJ (2005). Health workforce design for the 21st century. Aust Health Rev.

[2] Baum F, Begin M, Houweling TAJ, Taylor S (2009). Changes not for the fainthearted: reorienting health care systems towards health equity through action on the social determinants of health. Am J Public Health.

[3] Caughey GE, Vitry AI, Gilbert AL, Roughead EE (2008). Prevalence of comorbidity of chronic diseases in Australia. BMC Public Health..

[4] Wilson KM, Brady TJ, Lesesne C (2011). An organizing framework for translation in public health: the knowledge to action framework. Prev Chronic Dis.

[5] Armstrong BK, Gillespie JA, Leeder SR, Rubin GL, Russell LM (2007). Challenges in health and health care for Australia. Med J Aust.

[6] Williams RL, Johnson SB, Greene SM, Larson EB, Green LA, Morris A (2008). Signposts along the NIH roadmap for reengineering clinical research – Lessons from the Clinical Research Networks Initiative. Arch Intern Med.

[7] Morris ZS, Wooding S, Grant J (2011). The answer is 17 years, what is the question: understanding time lags in translational research. J R Soc Med..

[8] Sala A, Landoni P, Veganti R (2011). R&D Networks: an evaluation framework. Int J Technol Manag.

[9] Carswell P, Manning B, Long J, Braithwaite J (2014). Building clinical networks: a developmental evaluation framework. BMJ Qual Safe.

[10] Australian Government Department of Health and Ageing (2012). Strategic Review of Health and Medical Research in Australia – Consultation Paper Summary.

[11] Brownson RC, Royer C, Ewing R, McBride TD (2006). Researchers and policymakers: travelers in parallel universes. Am J Prev Med.

[12] Shonkoff JP (2000). Science, policy and practice: three cultures in search of a shared mission. Child Dev.

[13] University of Sydney (2013). Health and Medical Research Strategic Review.

[14] Cunningham FC, Ranmuthugala G, Plumb J, Georgiou A, Westbrook JI, Braithwaite J (2012). Health professional networks as a vector for improving healthcare quality and safety: a systematic review. BMJ Qual Saf.

[15] Armstrong R, Waters E, Dobbins M, Lavis JN, Petticrew M, Christensen R (2011). Knowledge translation strategies for facilitating evidence-informed public health decision making among managers and policy-makers. Cochrane Database Syst Rev..

[16] Trochim W, Kane C, Graham MJ, Pincus HA (2011). Evaluating translational research: a process marker model. Clin Trans Sci..

[17] Mold JW, Peterson KA (2005). Primary care practice based research networks: working at the interface between research and quality improvement. Ann Fam Med..

[18] Hall KL, Feng AX, Moser RP, Stokols D, Taylor BK (2008). Moving the science of team science forward: collaboration and creativity. Am J Prev Med.

[19] Cummings J, Kiesler S (2007). Coordination costs and project outcomes in multi university collaborations. Res Policy..

[20] Levac D, Colquhoun H, O’Brien K (2010). Scoping studies: advancing the Methodology. Imp Sci..

[21] Thyer BA (2002). Evidence-based practice and clinical social work. Evid Based Ment Health.

[22] Jarvenpaa SL, Staples DS (2000). The use of collaborative electronic media for information sharing: an exploratory study of determinants. J Strateg Inf Syst..

[23] Pilerot O, Limberg L (2011). Information sharing as a means to reach collective understanding: a study of design scholars’ information practices. J Doc.

